# Improved Symptom Profiles and Minimal Inflammation in IBS-D Patients Undergoing a Long-Term Low-FODMAP Diet: A Lipidomic Perspective

**DOI:** 10.3390/nu12061652

**Published:** 2020-06-02

**Authors:** Antonella Orlando, Valeria Tutino, Maria Notarnicola, Giuseppe Riezzo, Michele Linsalata, Caterina Clemente, Laura Prospero, Manuela Martulli, Benedetta D’Attoma, Valentina De Nunzio, Francesco Russo

**Affiliations:** 1 Laboratory of Nutritional Pathophysiology, National Institute of Gastroenterology “S. de Bellis” Research Hospital, 70013 Castellana Grotte (Ba), Italy; antonella.orlando@irccsdebellis.it (A.O.); giuseppe.riezzo@irccsdebellis.it (G.R.); michele.linsalata@irccsdebellis.it (M.L.); caterina.clemente@irccsdebellis.it (C.C.); lauraprospero87@gmail.com (L.P.); manuela.martulli@irccsdebellis.it (M.M.); benedetta.dattoma@irccsdebellis.it (B.D.); 2 Laboratory of Nutritional Biochemistry, National Institute of Gastroenterology “S. de Bellis” Research Hospital, 70013 Castellana Grotte (Ba), Italy; valeria.tutino@irccsdebellis.it (V.T.); maria.notarnicola@irccsdebellis.it (M.N.); valentinadx@hotmail.it (V.D.N.)

**Keywords:** dietetics, fatty acids, FODMAPs, inflammation, irritable bowel syndrome, lipidomic analysis, red blood cell membranes, symptom assessment

## Abstract

Given the link between the minimal inflammation underlying irritable bowel syndrome (IBS) and dietary treatments, considerable attention has focused on diets low in fermentable oligosaccharides, disaccharides, monosaccharides and polyols (FODMAPs). In this context, inflammatory patterns and lipidomic investigations may shed light on the pathophysiological mechanisms whereby a low-FODMAP diet (LFD) improves the IBS diarrhoea (IBS-D) variant. Thus, we investigated whether a long-term LFD induced changes in symptom profiles, anthropometric characteristics, inflammatory markers (C-reactive protein, cyclooxygenase-2, and prostaglandin E2) and erythrocyte-membrane fatty acid (FA) composition in IBS-D patients. Twenty IBS-D patients underwent a 90 day personalised LFD programme, and were regularly evaluated at scheduled visits. At the diet’s end, both IBS symptoms and anthropometric parameters were significantly improved. A significant decrease in prostaglandin E2 also accompanied these reductions. As for FAs, the putative inflammatory indicators, arachidonic acid (AA) levels and the AA/eicosapentaenoic acid ratio were significantly decreased. In conclusion, IBS-D patients following a controlled long-term LFD experienced improved symptom profiles and decreased inflammatory markers linked to FAs. Lipidomic data may be insightful for unravelling the molecular mechanisms associated with IBS-D pathophysiology.

## 1. Introduction

Irritable bowel syndrome (IBS) is a functional gastrointestinal (GI) disease that significantly affects patient quality of life. IBS is characterised by abdominal pain or discomfort, classically linked to changes in bowel habits. A high percentage (10%–15%) of the general population, mainly in Western, industrialised areas, suffer from IBS. IBS affects more females than males [1].

IBS development is a key factor for GI specialist referral. Pain severity and associated psychological distress (in some cases) are key determinants for patients seeking increased medical healthcare. An IBS diagnosis is still primarily based on specific GI symptom questionnaires, stool characteristics and the exclusion of organic GI diseases [2].

Based on stool characteristics, four IBS variants have been identified: diarrhoea (IBS-D), constipation (IBS-C), mixed (IBS-M), and undefined (IBS-U). IBS pathophysiology appears to involve, to varying degrees, low-grade inflammation, abnormal motility, modifications in intestinal barriers, alterations in gut–brain communications, psychosocial factors, increased GI fermentation and food intolerance [3,4].

Reports on self-perceived food intolerance suggests that high numbers of IBS patients (64%–89%) state that their symptoms are related to specific meals or foods [5]. Many patients modify their diets without professional counselling, potentially exposing themselves to prolonged nutritional deficiency [6]. Typically, foods are not considered the basis for intolerance, but rather as factors eliciting symptoms; therefore, they represent a major management pathway for many individuals. Thus, in recent years, much attention has been paid to specific dietary interventions to both improve symptom profiles and increase an often-low quality of life. In this framework, a diet low in fermentable oligosaccharides, disaccharides, monosaccharides and polyols (FODMAPs) can be used as a therapeutic approach to manage these symptoms [7].

FODMAPs represent a broad group of fermentable carbohydrates, including oligosaccharides (fructans and galactans), disaccharides (lactose), monosaccharides (fructose) and polyols (sorbitol and mannitol). These short-chain carbohydrates are poorly absorbed in the small intestine due to a lack of specific absorptive pathways, limited absorptive capacity or enzymatic deficiency, and are fermented in the colon [8].

An intriguing aspect of IBS is the link between the supposed minimal inflammation underlying IBS and dietary approaches. From an aetiological perspective, evidence suggests the existence of an inflammatory component, at least in well-defined IBS cases [9,10]. Previously, our group identified higher concentrations of inflammatory cytokines, e.g., interleukins (IL)-6 and IL-8, resistin and adiponectin in IBS-D patients when compared with healthy controls. Interestingly, these inflammatory cytokine levels were similar to those of patients with coeliac disease (CD) [10]. In this context, there is growing evidence to suggest that excessive fatty acids (FAs), i.e., saturated fatty acids (SFAs), monounsaturated fatty acids (MUFAs), polyunsaturated fatty acids (PUFAs) and endogenous trans-FAs, could play active roles in sustaining inflammatory processes [11]. Notably, such increases in FA levels may be related to diet [12,13].

Among these FAs, PUFAs affect inflammatory processes in both IBS-D patients and IBS animal models [14,15]. High omega-6/omega-3 PUFA ratios are implicated in a variety of inflammatory states, including inflammatory bowel disease [16]. In particular, omega-6 PUFAs exert pro-inflammatory reactions, whereas omega-3-PUFAs exert antioxidant effects. Arachidonic acid (AA) and eicosapentaenoic acid (EPA) represent the active biological forms of omega-6 and omega-3 PUFAs, respectively, and the AA/EPA ratio reflects inflammatory indices in patients with metabolic diseases [17,18]. Previously, we demonstrated that increased AA/EPA ratio was associated with more severe liver steatosis [19], suggesting that AA’s inflammatory effects contribute to liver injury pathogenesis.

The quality and quantity of accumulated lipids in erythrocyte membranes may be considered systemic nutritional ‘reporters’, resulting from interactions between genetic, metabolic and dietary factors [20].

Lipidomic analyses have been used to characterise specific FA profiles reflecting metabolic or chronic GI disorders, such as IBS [21]. Dietary habits are the prime determinants of FA composition [22,23]; thus, controlled nutritional interventions can restore normal cell-membrane lipidomic profiles.

Based on these observations, the main aims of this study were to (1) evaluate IBS-D patient symptom profiles, anthropometric characteristics and inflammatory pattern, by evaluating C-reactive protein (CRP), cyclooxygenase-2 (COX-2), prostaglandin E2 (PGE2), and erythrocyte-membrane FA composition in IBS-D patients, and (2) investigate whether a long-term low-FODMAP diet (LFD) induced changes in these parameters.

## 2. Materials and Methods

### 2.1. Patient Profile

Patients suffering from IBS-D in accordance with the Rome IV criteria [24] were recruited between January 2018 and September 2019 from among the outpatients of the Laboratory of Nutritional Pathophysiology—National Institute of Gastroenterology “S. de Bellis” Research Hospital.

Patients (18–65 years) underwent a GI visit and physical examination to confirm the presence of active symptoms resembling IBS-D (complained of for at least two weeks) and diarrhoea stool pattern. The visit was completed with the administration of the Gastrointestinal Symptom Rating Scale (GSRS) questionnaire [25]. Patients were asked to provide blood test results from within the previous three months (namely tests for liver function, thyroid function and reactive protein C), along with stool occult blood tests on three determinations, stool culture and a stool test for parasites. Recent gastroscopy and colonoscopy results were also requested.

Criteria of exclusion were: constipation, post-infectious IBS, giardiasis, pregnancy, previous abdominal surgery, metabolic and endocrine disorders, hepatic, renal, or cardiovascular disease, fever, intense physical activity, secondary causes of intestinal atrophy or a history of malignancy; antibiotic therapy or probiotic agents and other medications known to cause abdominal pain, consumption of selective serotonin reuptake inhibitors (SSRIs) or other antidepressant drugs; any consumption of drugs for treating IBS in the two weeks before the evaluation. To exclude CD, tissue transglutaminase and anti-endomysium antibodies were evaluated. Additionally, to avoid the possible presence of gluten-sensitive diarrhoea without CD that has been observed in IBS patients positive for HLA-DQ2 or HLA-DQ8, only the HLA-DQ2/HLADQ8-negative/negative IBS-D patients were recruited [26]. Before entering the study, patients were asked not to follow any diet that excessively limits certain nutrients (e.g., LFD, gluten-free or vegan diets).

Any reasons for study discontinuation were recorded in the case report form and included adverse events (specified), ineligibility to continue the study, withdrawal of consent, loss to follow-up or other causes.

All the subjects were compliant and were willing to participate in the study. Written informed consent was obtained from all the patients for blood testing and clinical data collection. This study was part of a research project approved by both the local scientific committee and the Institutional Ethics Committee of IRCCS Ospedale Oncologico di Bari—Istituto Tumori Giovanni Paolo II, Bari, Italy, Prot. N. 274/C.E. on 12 December 2017, and it is registered on http://www.clinicaltrials.gov (reg. Number NCT03423069).

### 2.2. Study Design

**Figure 1:**
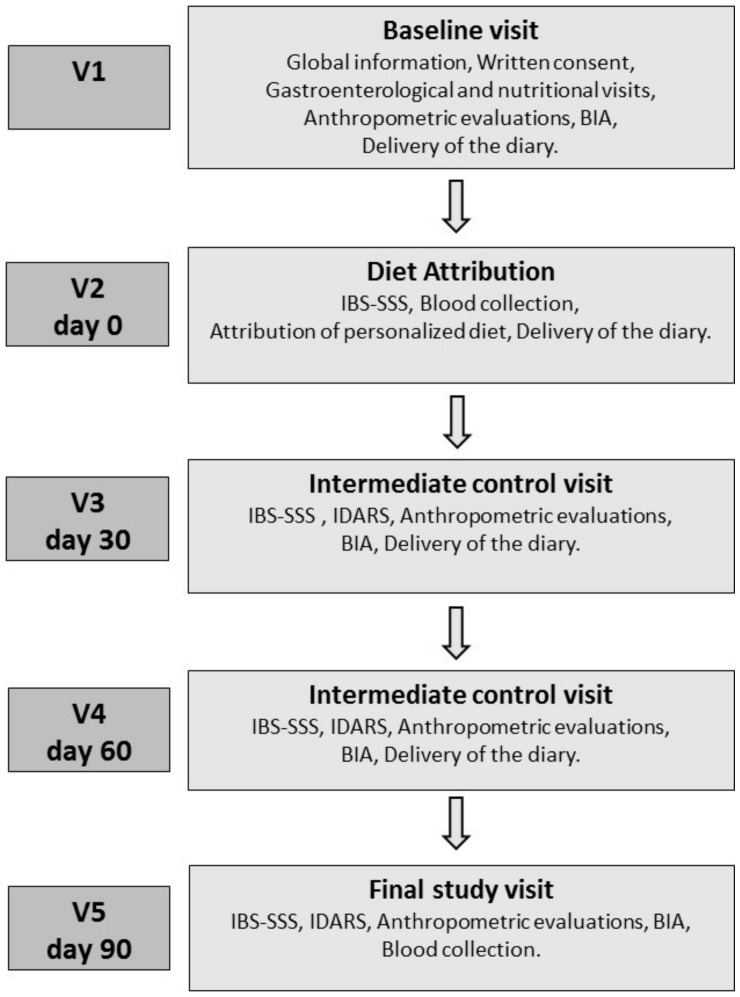
Schematic study drawing. BIA: bioelectrical impedance analysis; IBS-SSS: IBS Symptom Severity Scale; IDARS: IBS diet-adherence report scale.

Figure 1 schematises the study design, which consisted of five appointments. *Baseline Visit (V1):* During Visit 1, patients underwent a gastroenterological visit, received verbal and written information about the study and gave their informed consent. The patients were informed that the study aimed to evaluate the efficacy of a diet capable of alleviating IBS symptoms, and that it was to be followed for 90 days. However, patients were also informed that the diet was not intended to cure IBS or to remove all symptoms. All patients always met the same professionals, and the word “FODMAPs” was never used. Additionally, patients underwent an interview with qualified nutritionists to assess their lifestyle, dietary habits, physical activity, physiological status and possibly pathological conditions. The following anthropometric parameters were evaluated: weight, height, body mass index (BMI) and abdominal and waist circumferences. Bioelectrical impedance analysis (BIA) was used to measure resistance (Rz) and reactance (Xc) of human tissue by injecting a sinusoidal constant (800 µA) current at 50 kHz. The measurements were conducted as recommended by the guidelines of the European Society of Parenteral and Enteral Nutrition (ESPEN), under strictly standardised conditions [27] and using the same device (BIA 101, Akern SRL, Pontassieve, FI, Italy). All patients undergoing the BIA had been fasting for at least 4 h and had not ingested alcohol or performed intense physical activity in the previous 12 h. The phase angle (PhA, calculated as the arc tangent of the Xc/Rz ratio), body cell mass (BCM) and the body compartments, namely fat-free mass (FFM), fat mass (FM), total body water (TBW) and extracellular water (ECW), were calculated directly from Rz and Xc using specific software (Bodygram PLUS Software v. 1.0, Akern SRL, Pontassieve, FI, Italy) through medically validated algorithms. The patients eligible to participate in the study were invited to consume their usual diet and fill in a daily diary of their food habits until the next visit (V2). The diary included recording of the characteristics of the stool based on the Bristol stool form chart [28], intestinal habits, medications, physical activity and food habits to provide an estimate of daily energy intake and energy consumption.

*Diet Attribution (V2, Day 0):* Seven days after the baseline visit, patients returned to the clinic to complete the IBS Symptom Severity Scale (IBS-SSS) [29]. The IBS-SSS total symptom score required to enter the study was >125. Furthermore, the inclusion and exclusion criteria were revised again to include eating habits through evaluation of the daily food diary completed in the seven days preceding V2. During the visit, the patients underwent blood sampling for lipidomic analysis from the red-blood-cell membranes and to perform the analytical measurements. Patients enrolled in the study were asked to follow their personalised diet and were invited to fill a daily diary until the end of the nutritional intervention, in which they recorded the characteristics of their stool based on the Bristol stool form chart, intestinal habits, medications, physical activity and their food habits.

*Intermediate Control Visits (V3, Day 30; V4, Day 60):* During V3 and V4, the symptom and food questionnaires completed in the previous days were collected, and the patients received the new IBS-SSS and the questionnaire on adherence to the diet (IBS diet-adherence report scale—IDARS). This questionnaire consists of five questions on the adherence to dietary treatment with a score for each item ranging from one to five. A total score equal to or higher than 20 is representative of good adherence to the diet [30]. BIA and anthropometric measurements were also performed.

*Final Study Visit (V5, Day 90):* During V5, the symptom and food questionnaires completed in the previous days were collected, and the patients received the IBS-SSS and IDARS questionnaire. BIA and anthropometric measurements were also performed during V5. As at V2, during this visit, the patients underwent blood sampling for lipidomic analysis of the red-blood-cell membranes and to make the analytical measurements.

### 2.3. Symptom Profile

The symptom profile in IBS-D patients was studied by administering the IBS-SSS, a validated questionnaire for GI symptoms [29]. This scoring system is a global measure of five items describing the severity of IBS symptoms based on the visual analogue scale (VAS). The listed symptoms are the severity of abdominal pain, the frequency of abdominal pain, the severity of abdominal distension, dissatisfaction with bowel habits and the impact of symptoms on quality of life. Each symptom is described on a 100 point scale. A final item asks for the number of days out of ten in which the patient experiences abdominal pain, with the answer multiplied by 10 to create a metric between 0 and 100. The five elements are added together to provide a total score between 0 and 500. Scores indicated the cases as mild, moderate or severe at 75 to 175, 175 to 300, and >300, respectively. Healthy subjects have a score below 75, and patients scoring in this range can be considered in remission.

### 2.4. Assessment of Nutrient Intake

All patients had to complete a food diary every day, both before and during the assigned dietary intervention, to evaluate their daily energy intake and energy consumption. The diary included details of the quantities (expressed in grams) and the types of food consumed daily at breakfast, lunch, dinner and during snacks, as well as the type of physical activity and duration. Nutritionists examined food diaries completed before and during the nutritional intervention period. All data were recorded using appropriate software (Progetto Dieta v. 2.0—http://www.progettodieta.it) to obtain the daily energy intake and energy consumption expressed in kcal; the percentage and weight of daily proteins, carbohydrates and lipids; the amount of alcohol; and the weight of the dietary fibre.

### 2.5. Intervention Diet

A personalised LFD was assigned after examining the food diaries and during face-to-face individual counselling with the nutritionists in the V2. For all diet, the cut-off values of each FODMAP subgroup were applied by means of the published FODMAP table contents [31,32,33]. A diet is considered to be low in FODMAPs if it provides less than 3 g/day [34]. Dedicated software (Nutrigeo software 8.6.0.0, Progeo Medical, Centobuchi di Monteprandone (AP), Italy) was used to calculate the daily macronutrient intake according to a typical Mediterranean diet (50% carbohydrates, 30% lipids and 20% proteins). The diets were developed by matching the results of basal metabolism and daily energy consumption obtained from the population study with anthropometric data in order to assign adequate and personalised dietary regimens. Additionally, patients received a brochure with detailed information on permitted foods, which foods to avoid and which to reduce following the Monash University classification [35].

Nutritionists prepared a booklet for patients in the study with detailed information on where to buy specific products. An adequate intake of fibre was guaranteed by nutritionists, who also provided advice on how to cook without onion and garlic. Although not high in FODMAPs, the intake of alcohol was not recommended. In-between visits were performed every 30 days (specifically, at V3 and V4), at which patients had to provide a food diary to check compliance with the diet (IDARS). Lastly, patients could contact the nutritionists by phone during the diet period for any necessary information.

### 2.6. Analytical Measurements

A whole blood sample was taken from each IBS-D patient after 12 h of fasting by venous puncture. Blood samples were collected in vacutainer tubes containing ethylene–diamine–tetra-acetic acid (EDTA-K2) anticoagulant for FA analysis or with silica gel for routine analyses.

Routine biochemical analyses were conducted to evaluate the values of fasting serum total cholesterol (mg/dL), HDL (mg/dL), LDL:HDL ratio, triglycerides (mg/dL) and CRP (mg/dL). LDL values were calculated according to the Friedewald formula [36].

Serum COX-2 and PGE2 concentrations were measured in duplicate using commercially available sandwich enzyme-linked immunosorbent assay kits (catalogue number MBS164164 and catalogue number MBS700844, respectively; MyBioSource, San Diego, CA, USA).

### 2.7. Fatty Acids Analysis

FA extraction and lipid transesterification to fatty-acid methyl esters (FAME) were carried out using an automated protocol (Robot LNG-R1, Lipinutragen-Tecan, Bologna, Italy). Briefly, the whole blood in EDTA was centrifuged at 4000 × *g* for 5 min at 6 °C, the samples were inserted into the automated process for the separation of mature red blood cells and the plasma was removed. Erythrocytes at least three months old, isolated according to their density, were broken through an osmotic lysis process, and isolated membrane pellets were used to extract phospholipids using the Bling and Dyer method [37]. The organic layer was separated and dried using a centrifugal evaporator (Thermo Fisher Scientific, Waltham, MA, USA), and the FAME obtained were transesterified with potassium hydroxide (KOH)/methyl alcohol (MeOH) solution (0.5 mol/L).

After FAME extraction using n-hexane, esterified FAs were analysed using gas chromatography equipment with an auto-sampler, a split/splitless injector, FID detector and a hydrogen gas generator (Thermo Fisher Scientific, Milan, Italy) as previously described [38]. Quantification of FAME was performed using a mixture of standards (Supelco 37-Component FAME Mix, Sigma-Aldrich, Milan, Italy).

### 2.8. Statistical Analysis

All results are expressed as mean ± SEM. Paired Student’s *t*-test and Wilcoxon matched-pair signed-rank test were performed where appropriate. Friedman test with Dunn’s multiple comparison post-test was used to analyse the IDARS values at V3, V4 and V5.

Linear regression analysis was performed, considering the IBS-SSS difference before and after treatment as the dependent variable and FODMAP content, BMI, inflammation markers and FAs as independent variables in a stepwise regression procedure. For the regression, the explained variance (adjusted R square) was determined, and it was tested with the *F*-test. *t*-Values and their significance level were calculated to test the hypothesis that the contribution (the regression coefficient) of an entered variable significantly differed from zero.

All the differences were considered significant at a 5% level. A specific statistical package for exact nonparametric inference (2005 Stata Statistical Software Release 9; Stata Corp., College Station, TX, USA) was used.

## 3. Results

### 3.1. Patients and Symptom Profile

**Figure 2:**
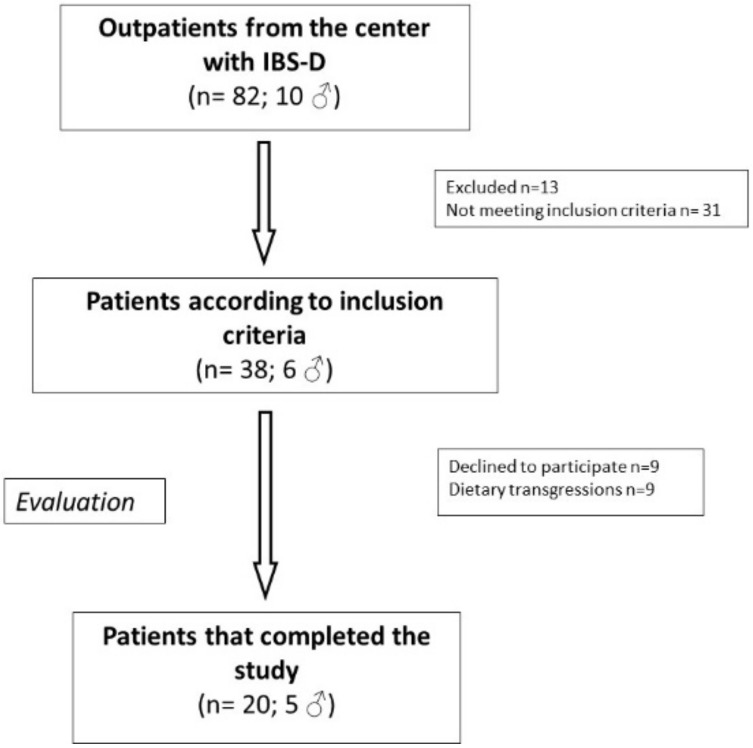
The flowchart of participant inclusion in the study. IBS-D: irritable bowel syndrome with prevalent diarrhoea.

Figure 2 shows the flow chart of patient selection. Eighty-two (72 F and 10 M) subjects suffering from IBS-D were recruited into this study. Of these patients, 13 were excluded for different reasons, 31 did not meet the inclusion criteria, 9 declined to participate (after assuming LFD for less than 30 days) because of difficulty following the diet and/or large time consumption not compatible with their occupation. The other nine were excluded for dietary transgressions. Finally, 20 IBS-D patients (5 men and 15 women; mean age = 45 ± 9.61 years) completed the study and followed the LFD for 90 days.

**Figure 3:**
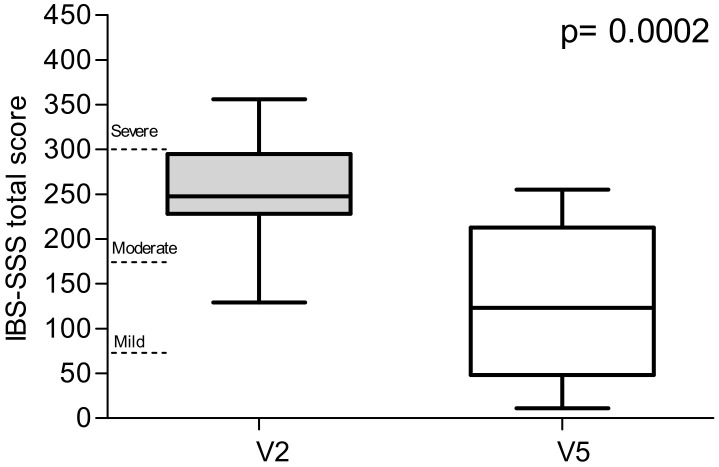
The total IBS Symptom Severity Scale (IBS-SSS) scores, recorded at V2 (diet attribution) and V5 (final study visit).

Figure 3 represents the total IBS-SSS scores. The value decreased significantly by 50.2% after 90 days of treatment (252.8 ± 14.1 vs. 125.9 ± 20.3; *p* = 0.0002). On the basis of the IBS-SSS total score at V2, 14 out of 20 patients were categorised as moderate cases and 5 as severe. Only one patient was a mild case. The proportion of patients defined as responders (IBS-SSS reduction ≥ 50) was 13/20 (65.0%).

When assessing the effect of the intervention on the individual items of the IBS-SSS score (Table 1), all items were significantly improved after the intervention. Additionally, after the LFD, the number of bowel movements per day was reduced at the end of the treatment period relative to baseline. In the IBS-SSS bowel habit items, the proportion of patients with a dominant Bristol stool form 5–7 reduced from 75% to 32% after the intervention.

### 3.2. Anthropometric Characteristics and Intervention Diet

The anthropometric characteristics of the patients at V1 (baseline visit) and after LFD intervention V5 (final study visit) are summarised in Table 2.

Significant decreases in weight, BMI and abdominal and waist circumferences were observed at V5 compared to V1. In addition, FM, FFM, TBW and ECW significantly reduced at V5 (final study visit) in comparison with V1 (baseline).

Table 3 shows the main daily nutritional information of patients at V2 and V5, respectively. A significant increase of both the protein and carbohydrate percentages at V5 in comparison with V2, was observed. In contrast, a significant reduction of lipid grams and percentage as well as the total FODMAP content compared to V2 occurred.

An example of the meals used in the LFD treatment is shown in Table 4.

Finally, the patients showed an excellent adherence to the LFD, as demonstrated by the total IDARS mean scores, which were always higher than 20 (Table 5).

### 3.3. Biochemical Parameters

Serum lipid concentrations and the main markers of systemic inflammation at the diet attribution (V2) and final study visit (V5) are reported in Table 6. No statistically significant difference was found in serum lipid concentrations between the two visits. As regards the markers of inflammation evaluated in the study, CRP and PGE2 levels, but not COX-2 ones, decreased at the end of the intervention (−23.5% and −21.1%, respectively), reaching statistical significance (*p* = 0.0005) in the case of PGE2.

### 3.4. Fatty Acids Profile

Table 7 shows the levels of the main fatty acids studied in the red-blood-cell membranes of the IBS-D subjects at V2 (diet attribution visit) and V5 (final study visit). After 90 days of dietary treatment, the AA levels were significantly reduced, consequently causing a significant reduction of AA/EPA ratio. Moreover, compared to the V2 values, a statistically significant decrease of the n-6 PUFAs/n-3 PUFAs ratio was present in the subjects after treatment. No changes in the levels of other FAs studied were observed.

### 3.5. Regression Analysis

Regression analysis showed that the change in IBS-SSS could be significantly explained by a linear combination of all the variables considered (i.e., FODMAP content, BMI, COX-2 and the AA/EPA ratio) (F = 6.93; df = 4; *p* = 0.005; adjusted R^2^ = 0.71) (Table 8). These data suggest that IBS symptoms should be considered as a sum of nutritional, biochemical and inflammatory abnormalities.

## 4. Discussion

In recent years, several studies have reported the effectiveness of a LFD in treating IBS patients [39,40,41,42]. In this context, we investigated whether a long-term LFD under constant nutritional control could positively affect patient symptom profiles, anthropometric characteristics, inflammatory patterns and erythrocyte-membrane FA composition in IBS-D patients. Nutritionist advice and supervision were fundamental during this study, as the potential limitations of a demanding LFD can be amplified in patients without professional help [43].

Enrolled patients were informed that the diet had potentially beneficial effects on GI symptoms. To avoid patient bias (extraneous online searches, advertising, etc.), a number of confidentiality measures were instigated; the diet was not referred to as LFD, but instead assigned the generic “diet X” in verbal and written communications. Similarly, the term ‘FODMAPs’ was not used, as this may have generated an unwanted placebo effect due to the extensive advertising of this diet, potentially resulting in undesired patient expectations. We also used the IBS-SSS questionnaire, which is a reliable and appropriate tool to investigate/catalogue IBS symptoms. After 90 days on the diet, total scores and single items (i.e., abdominal pain intensity and frequency, abdominal distension, dissatisfaction with bowel habits, general interference with life and stool frequency) were all significantly reduced. These observations supported the validity of a controlled long-term LFD in treating symptom profiles in IBS-D patients, even though a limitation of the study was the high number 18 of 38 (47%) of participants who either dropped out or were excluded for non-adherence. Potentially these participants either found the diet or the diet recording too challenging.

In contrast with other studies, our diet had a prolonged duration, i.e., 90 days instead of the usual four weeks. It was hypothesised that a long-lasting LFD was more reflective of GI symptoms and effects than a four week diet, and potentially mirrored natural fluctuations in symptom profiles in IBS-D patients, irrespective of medication. Associations between IBS-SSS score differences before and after treatment for FODMAP content, body mass index (BMI), COX-2 and AA/EPA ratio confirmed that reductions in IBS-SSS total scores could be explained by a linear combination of nutritional, anthropometric and inflammatory variables, acting synergistically.

As expected, the ingestion of all FODMAP classes was reduced. Patients adhered to and complied well with the LFD. This was in part thanks to the supplied study materials and continuous support from nutritionists. All subjects followed a personalised, balanced LFD, and this regimen led to remodulating the assumed macronutrient composition. Carbohydrates were significantly increased on the LFD, despite the drastic reduction in foods containing FODMAPs. This evidence emphasised the requirement for counselling services following this dietary approach, in order to avoid possible nutritional imbalances.

Anthropometric characteristics of IBS-D patients were also evaluated. Significant decreases in weight, BMI, abdominal and waist circumferences, FM, FFM, TBW and ECW were observed after LFD. These reductions were probably due to the restrictive nature of the diet and its duration. Weight loss and decreased BMI and FM were not research objectives. Notwithstanding, these factors represented inevitable consequences of a long-term personalised diet, which introduced a daily lipid intake based on a typical Mediterranean diet. Guerreiro et al. [44] reported that the “elimination phase” of a LFD leads to reduced body weight and BMI, following caloric restriction of some foods high in FODMAPs. Portion control is essential in a LFD, as larger portions can surpass safe thresholds, changing food from low to high FODMAP content [31].

Despite increased protein percentages after LFD, a statistically significant decrease in FFM was observed at the intervention’s end. This FFM decrease was due to significant TBW and ECW reductions at the end of the LFD. Moreover, no reductions were observed in BCM, which is the protein-rich compartment affected by catabolic states [27].

Reduced TBW and ECW values were accompanied by non-significant improvements in PhA. PhA is an indicator of cell-membrane integrity and water distribution between intracellular and extracellular compartments [45]. A low PhA can result from low cellular mass and quality, but also from fluid overload [46]. We observed decreases in TBW, ECW and FM, mainly as visceral fat, which we believe contributed to improvements in inflammatory parameters. There is a close link between hydration and inflammation; the body can send additional blood flow to inflammatory tissue, causing an increase in ECW [47,48].

Similarly, reductions in visceral fat, which produces large quantities of pro-inflammatory cytokines (e.g., TNF-*α* and IL-6), can improve inflammatory loads [49]. Overall, these findings agreed with the significant reductions in PGE2 serum levels accompanied by non-significant decreases in CRP and COX-2 in our study. This evidence strengthens the notion that IBS is a functional disorder, but with underlying abnormal immune function/activation [50]. If not present in all IBS patients, it is possible that in some IBS-D patients, dysbiosis, alterations in intestinal permeability [51] or miscellaneous environmental exposure may induce low-grade inflammation [9], affecting symptom profiles [50].

A key feature of our study design when compared to other study designs was diet duration, i.e., 90 days versus 28 days. This prolonged nutritional treatment allowed us to investigate FA composition in mature erythrocytes. Erythrocytes typically survive for four months in the blood; therefore, FA composition of red-blood-cell membranes may be representative of FA composition in bodily tissues [52,53].

It is accepted that cell-membrane lipidomic profiles are closely connected with dietary intake [19,54]. Uncorrected eating behaviours affect inflammatory processes, primarily sustained by an altered omega-6/omega-3 PUFA ratio. Imbalance in favour of omega-6 PUFAs is often associated with high levels of pro-inflammatory mediators and immune cell activation [55].

AA, which is derived from omega-6 PUFAs and enters the eicosanoid synthesis pathway, is believed to be a hallmark of inflammatory status in IBS patients; when compared with control subjects, increased plasma AA levels are often observed in IBS patients [52,56]. AA and its metabolites appear to be directly involved in intestinal motility, secretion and immunological functions in the gut, factors which are associated with IBS onset [16,57]. In this study, we observed that our personalised dietary intervention also reduced AA levels in red-blood-cell membranes in IBS-D patients.

At the V2 visit, all participants showed normal serum lipid and FA profiles in red blood cells, except for AA/EPA ratio. The lower AA percentage, and thus AA/EPA ratio, suggested that this dietary approach predisposed cells toward anti-inflammatory outcomes.

## 5. Conclusions

Our data demonstrated that combining lipidomic data with dietary assessments was useful in investigating the molecular mechanisms underlying symptom profile improvements in IBS-D patients following a prolonged and controlled dietary intervention. Our findings confirmed the existence of minimal inflammation in IBS-D; at the diet’s end, we observed significant decreases in PGE2 levels, linked to FAs such as AA and AA/EPA ratio. Lastly, our lipidomic approach not only generated nutritional indications for optimised membrane function, but it could also be used to search for novel biomarkers underpinning the clinical management of IBS patients.

## Figures and Tables

**Table 1:** IBS-SSS items of IBS-D patients recorded at V2 (diet attribution) and V5 (final study visit).

	V2 (*n* = 20)	V5 (*n* = 20)	*p*
Abdominal pain intensity	44.0 ± 5.1	16.4 ± 4.2	0.0016
Abdominal pain frequency	44.4 ± 6.8	17.5 ± 5.9	0.0001
Abdominal distension	46.5 ± 6.7	21.6 ± 3.8	0.0039
Dissatisfaction with bowel habits	56.7 ± 5.6	34.9 ± 6.1	0.0299
Interference with life in general	61.2 ± 3.6	35.5 ± 6.9	0.0040
Stool frequency	2.0 ± 0.2	1.1 ± 0.1	0.0002

Data are expressed as means ± SEM. *p*-Value was determined by Wilcoxon signed-rank test; differences were considered significant at *p* < 0.05; V2: diet attribution; V5: final study visit.

**Table 2:** Descriptive statistics of the anthropometric characteristics of the IBS-D subjects at V1 (baseline) and V5 (final study visit).

	V1 (*n* = 20)	V5 (*n* = 20)	*p*
Weight (kg)	65.84 ± 3.04	62.21 ± 2.90	<0.0001
Height (cm)	164.80 ± 2.44	164.40 ± 2.44	ns
BMI (kg/m2)	24.12 ± 0.84	22.90 ± 0.84	<0.0001
Abdominal circumference (cm)	87.25 ± 2.27	84.29 ± 2.21	<0.0001
Waist circumference (cm)	78.46 ± 2.63	74.92 ± 2.35	<0.0001
PhA (degrees)	5.80 ± 0.15	6.00 ± 0.15	ns
BCM (kg)	25.42 ± 1.31	25.35 ± 1.35	ns
FM (kg)	17.58 ± 1.51	14.81 ± 1.40	<0.0001
FFM (kg)	48.26 ± 2.18	47.44 ± 2.14	0.0039
TBW (L)	35.30 ± 1.63	34.67 ± 1.59	0.0079
ECW (L)	16.50 ± 0.75	15.90 ± 0.64	0.0267

BMI: body mass index; PhA: phase angle; BCM: body cell mass; FM: fat mass; FFM: fat-free mass; TBW: total body water; ECW: extracellular water. Data are expressed as means ± SEM. *p*-Value was determined by Student’s paired *t*-test; differences were considered significant at *p* < 0.05; ns: not significant. V1: baseline; V5: final study visit.

**Table 3:** Descriptive statistics of the main daily nutritional information of IBS-D subjects at V2 (diet attribution) and V5 (final study visit).

	V2 (*n* = 20)	V5 (*n* = 20)	*p*
Energy consumption (kcal)	2073 ± 93.76	2067 ± 93.11	ns
Energy intake (kcal)	2046 ± 180.90	1830 ± 127.50	ns
Basal metabolism (kcal)	1507 ± 42.37	1522 ± 45.05	ns
Proteins (g)	77.75 ± 6.45	88.50 ± 5.44	ns
Proteins (%)	15.79 ± 0.42	19.50 ± 0.21	<0.0001
Lipids (g)	87.46 ± 10.41	60.35 ± 4.29	0.0387
Lipids (%)	36.42 ± 1.23	29.70 ± 0.22	<0.0001
Carbohydrates (g)	234.90 ± 16.01	247.20 ± 17.86	ns
Carbohydrates (%)	47.23 ± 1.26	50.54 ± 0.32	0.0156
Alcohol (%)	0.77 ± 0.29	0.26 ± 0.15	ns
Dietary fibre (g)	18.29 ± 1.03	17.15 ± 1.29	ns
Total FODMAPs (g/day)	20.73 ± 1.12	3.27 ± 0.10	<0.0001

Data are expressed as means ± SEM; *p*-Value was determined by Student’s paired *t*-test; differences were considered significant at *p* < 0.05; ns: not significant. V2: diet attribution; V5: final study visit. FODMAPs: Diet low in fermentable oligosaccharides, disaccharides, monosaccharides and polyols.

**Table 4:** Example of a personalised LFD for a patient with an approximate energy expenditure of 1800 kcal/day.

Meal	LFD (Total FODMAPs: 3.51 g/day)
Breakfast	Tea (200 g) + gluten-free biscuits (80 g)
Mid-morning snack	Banana (100 g)
Lunch	Gluten-free pasta (130 g) + courgette (75 g) + prawn peeled (60 g) + arugula (150 g)
Afternoon snack	Raspberries (125 g)
Dinner	Beef (130 g) + tomatoes (150 g)
During the day	Gluten-free bread (130 g) + virgin olive oil (6.5 teaspoons)

LFD: Low-FODMAPs diet.

**Table 5:** Descriptive statistics of IDARS of IBS-D subjects at V3 (intermediate control visit) V4 (intermediate control visit) and V5 (final study visit).

	V3 (*n* = 20)	V4 (*n* = 20)	V5 (*n* = 20)	*p*
IDARS	22.75 ± 04	23.06 ± 0.5	22.88 ± 0.5	ns

IDARS: IBS diet-adherence report scale; *p*-value was determined by Friedman test with Dunn’s multiple comparison post-test; ns: not significant; V3 and V4: intermediate control visits; V5: final study visit.

**Table 6:** Descriptive statistics of the serum lipid concentrations and the main markers of systemic inflammation of the IBS-D subjects at V2 (diet attribution) and V5 (final study visit).

	V2 (*n* = 20)	V5 (*n* = 20)	*p*
Total cholesterol (mg/dL)	184.20 ± 7.74	179.30 ± 7.46	ns
LDL (mg/dL)	109.70 ± 6.33	106.70 ± 6.71	ns
HDL (mg/dL)	57.85 ± 2.51	55.15 ± 2.71	ns
LDL:HDL ratio	1.93 ± 0.12	1.97 ± 0.13	ns
Triglycerides (mg/dL)	84.35 ± 7.57	86.90 ± 10.40	ns
CRP (mg/dL)	0.17 ± 0.04	0.13 ± 0.01	ns
COX-2 (u/L)	10.30 ± 1.18	10.19 ± 1.13	ns
PGE2 (pg/mL)	36.71 ± 7.86	28.95 ± 7.46	0.0005

LDL: low-density lipoprotein; HDL: high-density lipoprotein; CRP: C-reactive protein; COX-2: cyclooxygenase-2; PGE2: prostaglandin E2. Data are expressed as means ± SEM. *p*-Value was determined by Student’s paired *t*-test; differences were considered significant at *p* < 0.05; ns: not significant. V2: diet attribution; V5: final study visit.

**Table 7:** Mean percentage of red-blood-cell membrane fatty acids in IBS-D patients at V2 (diet attribution) and V5 (final study visit).

Red-Blood-Cell Membrane Fatty Acids	V2	V5	*p*	n.v. (% rel.)
	(*n* = 20)	(*n* = 20)		
***SFAs***				
C16:0 Palmitic acid	19.19 ± 0.52	21.50 ± 0.75	ns	17–27
C18:0 Stearic acid	14.82 ±0.42	15.46 ± 0.91	ns	13–20
***MUFAs***				
C16:1*n*7 Palmitoleic acid	0.51 ± 0.13	0.60 ± 0.35	ns	0.2–0.5
C18:1*n*9 Oleic acid	13.64 ± 0.55	14.26 ± 0.59	ns	9–18
C18:1*n*7 Vaccenic acid	1.17 ± 0.06	1.19 ± 0.05	ns	0.7–1.3
***PUFAs***				
C20:4*n*6 Arachidonic acid (AA)	16.32 ± 0.62	14.58 ± 0.34	0.037	13–17
C20:5*n*3 Eicosapentaenoic acid (EPA)	0.86 ± 0.08	0.82 ± 0.21	ns	0.5–0.9
C22:6*n*3 Docosahexaenoic acid (DHA)	5.23 ± 0.4	5.62 ± 0.55	ns	5–7
***Total FAs***				
SFAs	43.52 ± 0.77	45.30 ± 1.88	ns	30–40
MUFAs	20.1 ± 0.84	20.66 ± 0.72	ns	13–23
PUFAs	35.07 ± 1.3	33.62 ± 2.32	ns	28–39
***FA indexes***				
*n*-6 PUFAs/*n*-3 PUFAs ratio	3.91 ± 0.28	3.28 ± 0.16	0.048	3–4.5
AA/EPA ratio	23.25 ± 2.43	17.04 ± 1.0	0.025	<15

Data are expressed as means ± SEM; *p*-value was determined by Student’s paired *t*-test; differences were considered significant at *p* < 0.05; ns: not significant; n.v.: normal values; V2: diet attribution; V5: final study visit; SFAs: saturated fatty acids; MUFAs: monounsaturated fatty acids; PUFAs: polyunsaturated fatty acids; FAs: fatty acids.

**Table 8:** Regression analysis between IBS symptom score and anthropometric, nutritional and inflammatory variables.

Parameters	*β*	Std. Error (*β*)	*p*	95% CI
FODMAP content	10.28	4.46	0.042	1.54–19.02
BMI	87.83	20.15	0.001	48.33–127.33
COX-2	34.04	10.73	0.009	13.01–51.07
AA/EPA ratio	4.17	1.55	0.021	1.13–7.21

All variables were calculated as the difference before and after treatment. Linear regression analysis was performed, considering the IBS-SSS as the dependent variable and the other variables as independent variables. BMI: body mass index; COX-2: cyclooxygenase-2; AA/EPA ratio: arachidonic acid to eicosapentaenoic acid ratio.
